# Relative and normalized iodine concentrations derived from photon counting computed tomography and their correlation with tumor grade and Ki67 in pancreatic neuroendocrine neoplasia: A pilot study

**DOI:** 10.1111/jne.70137

**Published:** 2026-02-12

**Authors:** Marwin‐Jonathan Sähn, Simon Waltermann, Jonas Ottemöller, Raihanatou Diallo‐Danebrock, Julius Henning Niehoff, Marcel Bähr, Berthold Gerdes, Jan Borggrefe, Nehara Begum, Alexey Surov

**Affiliations:** ^1^ Department of Diagnostic and Interventional Radiology, Neuroradiology and Nuclear Medicine Johannes Wesling University Hospital, Ruhr‐University‐Bochum Minden Germany; ^2^ Department of Pathology Johannes Wesling University Hospital, Ruhr‐University‐ Bochum Minden Germany; ^3^ Department of General, Visceral, Thoracic and Endocrine Surgery, ENETS‐Center of Excellence Johannes Wesling University Hospital, Ruhr‐University‐ Bochum Minden Germany

**Keywords:** Ki67, neuroendocrine tumors, photon counting computed tomography

## Abstract

Neuroendocrine neoplasms are a rare and complex tumor entity, among which pancreatic neuroendocrine neoplasms generally display a more aggressive behavior. Despite a notable stage migration towards lower stages at initial diagnosis, the incidence of pancreatic neuroendocrine neoplasms is rising. In recent publications, iodine concentration derived from dual energy computed tomography was explored as a potential biomarker for pancreatic neuroendocrine neoplasia tumor grade and Ki67. However, methodologies exhibited significant variability, and reported outcomes were ambiguous, ranging from weak correlations to strong predictive performance in complex multivariate analyses. With the advent of photon counting computed tomography and improved technical capabilities, this study revisits the topic and aims to provide evidence for tumor characterization using photon counting computed tomography‐derived iodine concentration in pancreatic neuroendocrine neoplasms. Iodine concentration in neuroendocrine pancreatic primaries was analyzed in the portal venous phase regarding correlation with histopathological tumor grade and Ki67. Iodine concentration was normalized to aortic iodine concentration (normalized iodine concentration), as well as calculated relative to unaffected pancreatic tissue (relative iodine concentration). Correlations were analyzed using Spearman's rank correlation, and mean concentrations were analyzed using Mann–Whitney *U* test. Eighteen cases with pancreatic neuroendocrine neoplasms were included. Relative Iodine concentration exhibited a strong and statistically significant correlation with tumor grade (*ρ* = 0.54, *p* = 0.02) and Ki67 (*ρ* = 0.53, *p* = 0.02). Mean relative iodine concentration was higher in high‐grade tumors (*p* = 0.02). Normalized iodine concentration showed weak, non‐significant correlations with tumor grade (*ρ* = 0.33, *p* = 0.18) and Ki67 (*ρ* = 0.30, *p* = 0.22). Mean normalized iodine concentration did not differ significantly between low‐grade and high‐grade pancreatic NEN. Our preliminary results show that photon counting computed tomography derived iodine concentration and especially relative iodine concentration is a potential biomarker for tumor grade and Ki67 prediction with strong, statistically significant correlations in untreated pancreatic neuroendocrine neoplasms. The method is non‐invasive, requires little to no additional resources and may support early, evidence‐based therapeutic decisions even before primary tumor biopsy.

## 1 INTRODUCTION

Neuroendocrine neoplasia (NEN) is a heterogeneous group of malignancies with diverse biologic and clinical behaviors and prognoses depending on primary tumor site, tumor grading, and pathologic features. Most NEN originate from the lung and the gastrointestinal tract, with rising incidence of especially primary pancreatic NEN during the last decades.^1^ This rise in incidence, however, was accompanied by a notable stage migration towards lower tumor stages upon initial diagnosis.^1^ Hepatic metastases are most common.^2^ Pancreatic NEN (PanNEN) are graded into neuroendocrine tumor (NET) categories 1–3 and are distinguished from neuroendocrine carcinomas (NEC).^3^

Improved diagnostic with cross‐sectional imaging has led to increasing detection of small pancreatic lesions. Currently, an arterial hyperenhancement in CT‐scan and positivity in somatostatinreceptor PETimaging suggest a well‐ or moderately differentiated PanNEN. The risk of lymph node metastases increases with the size of the primary tumor. PanNEN up to 2 cm can be treated by watchful waiting if no functional activity and no other risk factors (rapid tumor growth, high Ki67% or locoregional spread) are present. A high‐profile, prospective international study (ASPEN‐Study) is currently investigating this strategy.^4^ Tumor grading involves analyzing mitotic rate and Ki67 proliferation index (Ki67).^3^ Ki67 was previously reported to outperform mitotic index in predicting prognosis.^5^ Tumor characterization in clinical routine typically requires tissue sampling (e.g., biopsy of metastases or resection specimen). The use of readily available information at the earliest possible stage would therefore be desirable. Due to the risk of complications (e.g., pancreatitis) and the technical challenges of puncturing small lesions, histological grading of the tumors is often not feasible in small incidental lesions suspicious of PanNEN. A correlation between cross‐sectional imaging parameters and tumor grade could close the gap in the initial risk stratification and follow‐up and may support the decision regarding the optimal timing of surgical resection.

Quantitative imaging biomarkers (QIBs), particularly in oncology, have recently been subject to extensive research.^6^ A QIB is any measurable parameter in a given imaging modality that predicts or reflects a clinically relevant state.^7^ Examples include coronary artery calcification scores in CT, standardized uptake value (SUV) in PET and diffusion‐weighted imaging/apparent diffusion coefficient (DWI/ADC) in MRI, for example, to monitor clinical status regarding characterization of breast cancer, lung cancer and renal masses.^8^, ^9^, ^10^ QIBs can be deployed, for example, to monitor tumor progression, regression or stable disease.^11^

In recent publications, iodine concentration and iodine maps have been shown to improve characterization of pancreatic carcinoma and prediction of chemotherapy response in pancreatic cancer.^12^, ^13^, ^14^ Wang et al. reported that iodine concentration in portal venous phase in dual‐layer spectral energy CT (DLCT; a DECT implementation) may differentiate between G1/2 NET and G3 NET/NEC with an AUC of 0.897.^15^

Cruz‐Hernandez et al. and Li et al. correlated DECT‐derived iodine concentrations in arterial phase (both normalized and relative measures) in PanNEN with tumor grading and Ki67.^16^, ^17^ Cruz‐Hernandez et al. reported weak correlations while Li et al. reported respectable AUC values for a multiparametric approach to stratify low‐grade from high‐grade neoplasms.^16^, ^17^

Photon‐counting computed tomography (PCCT) has emerged as an innovative imaging modality that addresses some limitations of conventional DECT.^18^ Energy‐integrating detectors (EIDs) used in DECT measure the total incoming energy, whereas photon‐counting detectors measure the energy of individual x‐ray photons.^18^ Among other advantages, PCCT offers improved spatial resolution, improved contrast‐to‐noise ratio, and enhanced spectral imaging capabilities.^18^

With the advent of PCCT and improved technical possibilities, this study revisits the role of iodine concentration and aims to provide evidence for tumor characterization using PCCT‐derived iodine concentration in PanNEN.

## 2 METHODS

This retrospective study was approved by the Ethics Committee of the Faculty of Medicine, Ruhr‐University Bochum (2021‐827).

### 2.1 Cohort recruitment

A hospital‐wide database query was performed to identify patients with pancreatic NEN.

### 2.2 Inclusion criteria were

- Patient with histopathologically confirmed PanNEN with available data regarding tumor grading and Ki67;
- PCCT staging including at least portal venous contrast phase

### 2.3 Exclusion criteria were

- Pancreatic tumors other than NEN:
- Insufficient image quality (e.g., artifacts);
- No image acquisition during the portal venous contrast phase.

**FIGURE 1:**
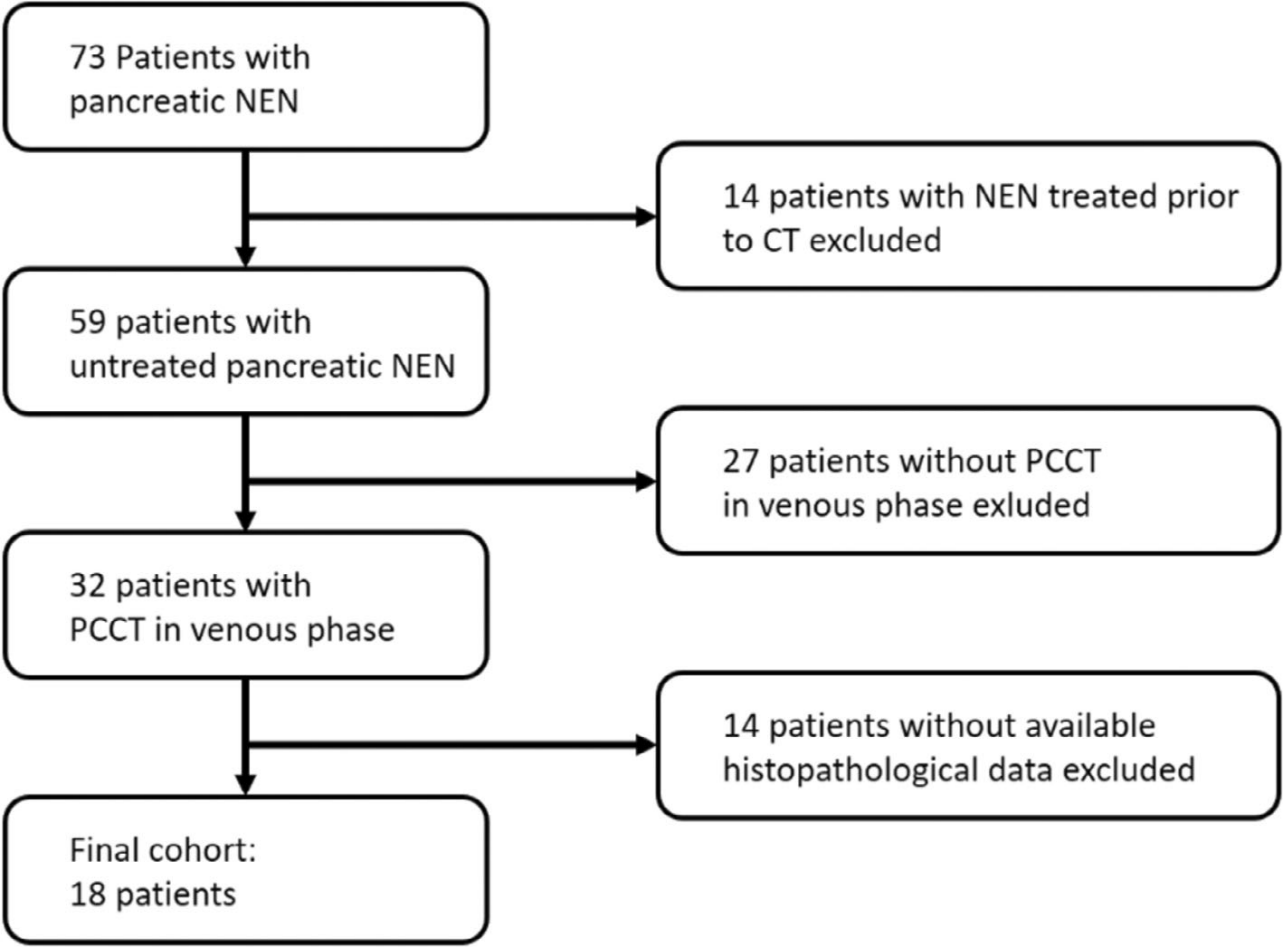
Patient recruitment algorithm flowchart. Patients with PCCT imaging performed from July 2022 to February 2025 were included.

Figure 1 depicts the patient recruitment algorithm and the numbers of included and excluded patients.

### 2.4 Image acquisition

All CT scans were performed using the Neoatom Alpha scanner (NAEOTOM Alpha, software version VB80B, Siemens Healthineers, Erlangen, Germany). Examinations were performed in supine positioning, with elevated arms. Detailed technical information can be found in the Supporting Information.

Contrast agent was injected i.v. at 2.4 mL/s until a dose of 1 mL/kg of patient body mass was achieved, followed by a chaser bolus of 40 mL sterile sodium chloride solution (NaCl 0.9%). PCCT scans were performed 60 seconds after a threshold value of 100 HU in the descending aorta was exceeded (bolus tracking).

### 2.5 Image analysis

**FIGURE 2:**
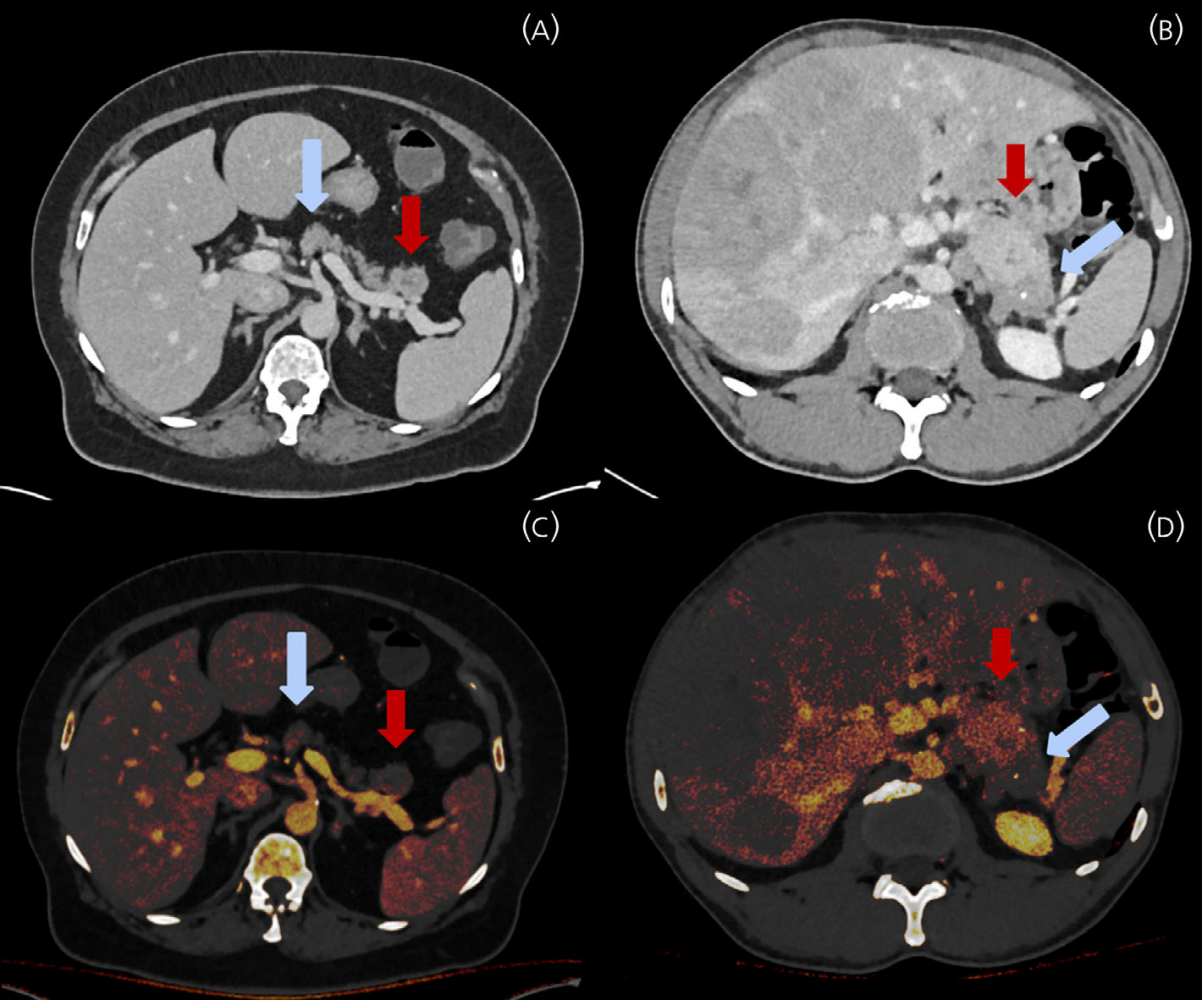
Representative images of hybrid iodine maps in patients with pancreatic NET corresponding to histopathological specimens in Figure 3. Figure 2A depicts a pancreatic neuroendocrine tumor (G1) within the pancreatic tail with Ki67 proliferation rate of 1%, whereas Figure 2B depicts a neuroendocrine tumor (G3) with Ki67 proliferation rate of 39% in venous contrast phase in computed tomography. Pancreatic neuroendocrine tumors are marked with red arrows while surrounding pancreatic tissue is marked with teal arrows. Figure 2C,D depicts corresponding iodine map superimposed on a monoenergetic virtual non contrast (VNC) slice. Tumor iodine concentration in venous phase is hardly elevated compared to surrounding pancreatic tissue in Figure 2C (G1), whereas a clear elevation of tumor iodine concentration can be observed in Figure 2D (G3). Multiple hepatic metastases are depicted in Figure 2B,D as well, though iodine concentration seems to diverge.

Iodine concentration measurements were performed in hybrid iodine maps by two radiologists (2 years of experience and 6 years of experience in colorectal imaging, respectively) in consensus and blinded to histopathological findings. Exemplatory images are depicted in Figure 2.

Polygonal regions of interest (ROIs) were placed within PanNEN in the axial slice with maximal tumor extension. To minimize partial volume effects, readers were instructed to maintain a border margin of at least 4 mm. ROIs were also placed within the descending aorta and unaffected pancreatic parenchyma.

### 2.6 TNM and histopathological data

**FIGURE 3:**
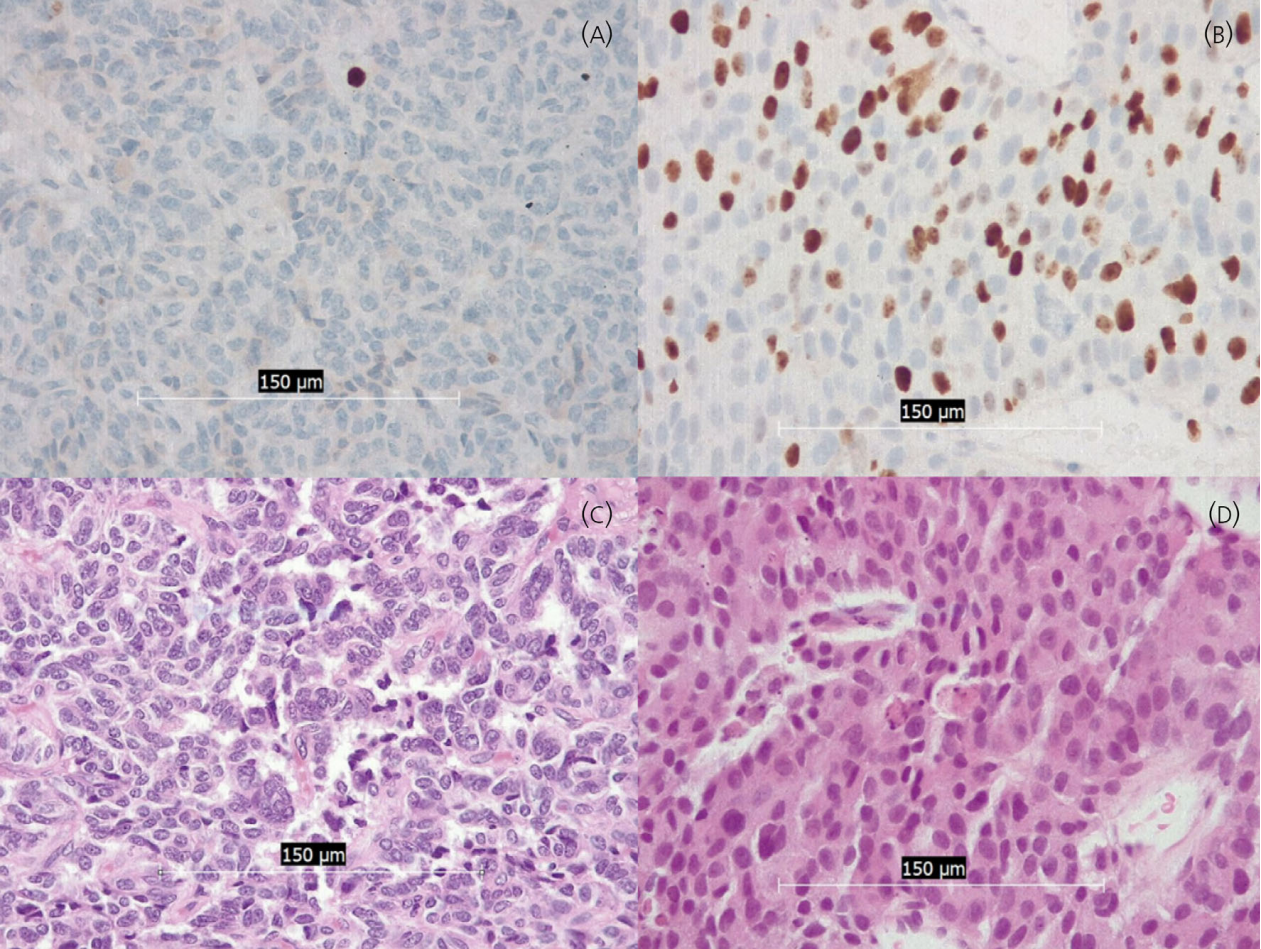
Exemplary histopathological specimens corresponding to PCCT‐derived images in Figure 2. Figure 3A depicts a pancreatic neuroendocrine tumor (G1) within the pancreatic tail with Ki67 proliferation rate of 1% (400×). Figure 3B depicts a pancreatic neuroendocrine tumor (G3) within the pancreatic tail with Ki67 proliferation rate of 39% (400×). Figure 3C,D depicts corresponding tumor tissue in hematoxylin and eosin (400×).

The following tumor features were analyzed: tumor grade, lymph node involvement and distant organ metastases, as well as Ki67‐associated proliferation rate (%) for PanNEN. Primary histopathological diagnosis was performed on hematoxylin–eosin (H&E) stained slides at 200x and 400x magnification. Ki67 was determined by immunohistochemistry. Exemplatory images are depicted in Figure 3. Examinations were performed by a board‐certified pathologist with 20 years of clinical experience.

### 2.7 Statistical analysis

Relative iodine concentration (RIC) and normalized iodine concentration (NIC) were calculated as follows:^17^

- RIC = Mean lesion iodine concentration/mean unaffected pancreatic parenchyma iodine concentration;
- NIC = Mean lesion iodine concentration/mean aortic iodine concentration

Spearman's rank correlations were calculated for iodine concentration (IC), RIC, and NIC with respect to tumor grade and Ki67. Mean values, variances, *F* tests, medians and interquartile ranges were calculated.

Mann–Whitney *U* tests, as well as Student's *t* tests for homoscedastic, unpaired data were performed to compare RIC/NIC between low‐grade (G1‐2, corresponding to KI67 ≤20%) and high‐grade (G3‐4 corresponding to Ki67 >20%) PanNEN. All *p*‐values were calculated two‐tailed. A 95% confidence level was chosen as a threshold for statistical significance.

Intraclass correlation coefficients and corresponding 95% confidence intervals were calculated for RIC, NIC and IC.

## 3 RESULTS

A total of 18 patients (12 female, 6 male) with PanNEN who underwent PCCT staging from May 2022 until February 2025 in our clinic were included in this study (Figure 1). Mean age was 70.8 years for men and 60.8 years for women (Table 1). Mean Ki67 was below the 20% threshold for both men (13.5%) and women (7%). Pancreatic NET had metastasized in four men and seven women (66.7% and 58.3%, respectively). No PanNEN was hormonally active.

**TABLE 1:** Demographic information and Ki67/ tumor grade distribution.

Demographic information	
Patient sex	12/18 (66.7%) female
Age (years)	64.2 ± 15.3
Mean Ki67 (%)	9.2% ± 10.3%
Metastases	11/18 (61.1%)
Hormonally active	0 (0%)

RIC correlated strongly and statistically significantly with both Ki67 and tumor grade, yielding a Spearman's rho (*ρ*) of 0.533 (*p* = 0.023) and 0.54 (*p* = 0.021), respectively. ICC for RIC was 0.827 (95% CI: 0.593–0.932).

**FIGURE 4:**
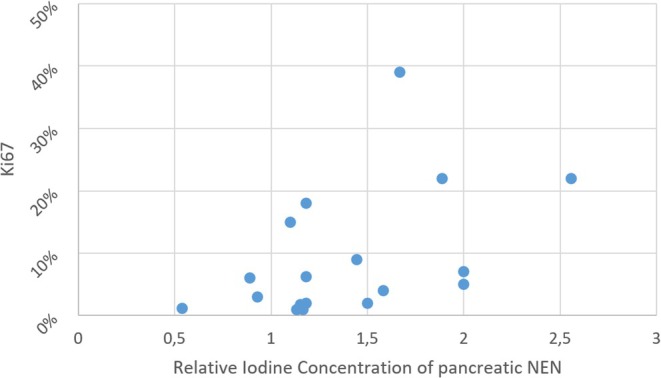
Scatter plot of tumor RIC relative to Ki67.

A scatter plot of source data is provided in Figure 4.

Mann–Whitney *U* test revealed statistically significant differences in RIC between high grade (G3/4) and low grade (G1/2) PanNEN, 2.037 versus 1.266 (*p* = 0.028), respectively.

NIC demonstrated mild, non‐significant correlations with that did not reach the 95% confidence level with Ki67 and tumor grading, yielding a Spearman rho (*ρ*) of 0.302 (*p* = 0.224) and 0.328 (*p* = 0.183), respectively. ICC for NIC was 0.717 (0.400–0.883).

**FIGURE 5:**
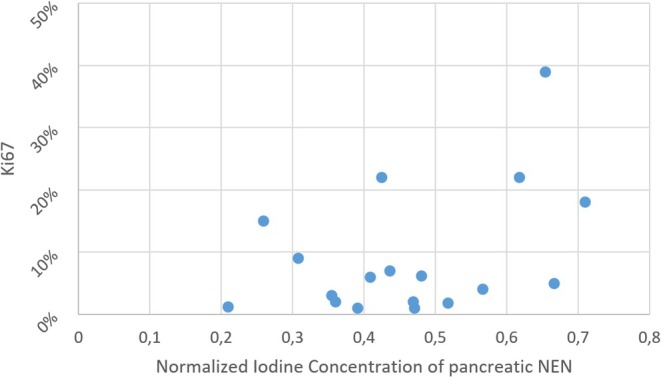
Scatter plot of tumor NIC relative to Ki67.

A scatter plot of source data is provided in Figure 5.

The Mann–Whitney *U* test yielded no statistically significant differences in NIC between high‐grade (G3/4) and low‐grade (G1/2) PanNEN, with an average NIC value of 0.565 and 0.44, respectively.

IC did not correlate with KI67 and tumor grade, with Spearman's rho (*ρ*) of −0.113 (*p* = 0.654) and −0.010 (*p* = 0.968), respectively. ICC for IC was 0.697 (0.365–0.874).

**FIGURE 6:**
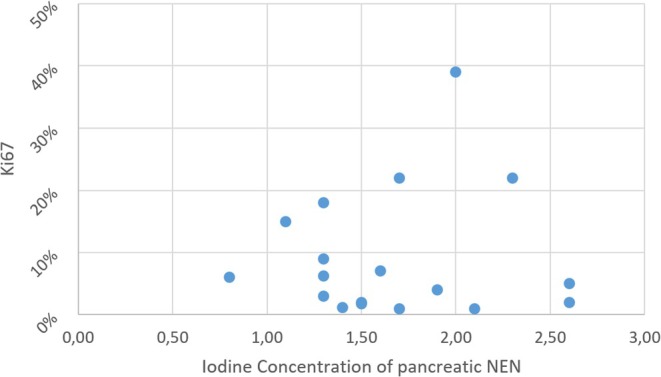
Scatter plot of tumor IC relative to Ki67.

A scatter plot of source data is provided in Figure 6.

Mean IC was 2.0 in high‐grade and 1.6 in low‐grade PanNEN, without statistically significant differences. Details are provided in Table 2.

**TABLE 2:** Statistical results of RIC and NIC, as well as IC and their respective influence on / correlation with Ki67 and tumor grade.

Statistical results					
RIC		NIC		IC	
Spearman correlation					
RIC	*ρ* = 0.533	NIC	*ρ* = 0.302	IC	*ρ* = −0.113
vs. Ki67	*p* = 0.023	vs. Ki67	*p* = 0.224	vs. Ki67	*p* = 0.654
RIC	*ρ* = 0.540	NIC	*ρ* = 0.328	IC	*ρ* = −0.010
vs. tumor grade	*p* = 0.021	vs. tumor grade	*p* = 0.183	vs. tumor grade	*p* = 0.968
Location parameters					
Mean RIC High grade	2.037	Mean NIC High grade	0.565	Mean IC High grade	2.000 mg
Mean RIC Low grade	1.266	Mean NIC Low grade	0.440	Mean IC Low grade	1.600 mg
Variance High grade	0.214	Variance High grade	0.015	Variance High grade	0.090 mg
Variance Low grade	0.153	Variance Low grade	0.019	Variance Low grade	0.261 mg
*F* test	*p* = 0.558	*F* test	*p* = 0.950		*p* = 0.571
Median RIC High grade	1.889	Median NIC High grade	0.565	Median IC High grade	2.000 mg
IQR high grade	0.333	IQR high grade	0.096	IQR high grade	0.150 mg
Median RIC Low grade	1.182	Median NIC Low grade	0.438	Median IC Low grade	1.500 mg
IQR low grade	0.333	IQR low grade	0.131	IQR low grade	0.450 mg
*U* test (high grade vs. low grade)					
*U* test RIC	*p* = 0.028	*U* test NIC	*p* = 0.214	*U* test IC	*p* = 0.124
Intraclass correlation coefficient					
RIC	0.827	NIC	0.717	IC	0.697
95% Confidence interval	0.593 to 0.932	95% Confidence interval	0.400 to 0.883	95% Confidence interval	0.365 to 0.874

## 4 DISCUSSION

With the relatively recent clinical availability of PCCT, this is, to our knowledge, the first study investigating PCCT‐derived iodine concentration and its derivatives RIC and NIC with respect to their correlation with Ki67 as an established prognostic biomarker in PanNEN, as well as tumor grade.

There is considerable evidence for tumor‐specific quantitative imaging biomarkers. For example, ADC in diffusion weighted MRI and SUV in PET imaging have been reported to have promising correlation with tumor malignancy, particularly in breast cancer.^19^ In this context, SUV and ADC have even been reported to predict molecular subtypes of breast cancer.^20^ ADC has also been reported to predict the efficacy of radiotherapy in prostate cancer and to serve as an imaging biomarker for diagnosis and treatment response in head and neck cancers.^21^

Among other benefits, CT‐derived iodine concentration has been shown to contain information that correlates with angiogenesis and prognosis in lung cancer.^22^ The previous literature has also suggested that iodine concentration has prognostic value in differentiating clear cell from papillary renal cell carcinoma.^23^ Iodine concentration can also predict rectal cancer response to neoadjuvant radiochemotherapy.^24^ Iodine concentrations have been reported to enable monitoring of immune checkpoint inhibitor response in metastases of melanoma and renal cell carcinoma.^25^ Chen et al. found iodine concentrations to be helpful in identifying lymph node metastases.^26^

In recent literature on NEN, results have been heterogenous, reporting weak to no significant correlation of iodine concentration derived parameters with Ki67,^17^ as well as highly promising findings suggesting that DECT‐derived iodine concentration or its normalized derivatives (e.g., RIC/NIC) possess sufficient predictive value to differentiate between a G1/2 PanNEN from G3 NET/NEC with an AUC of 0.897 in a multiparametric setting.^15^ More recently, other authors reported that iodine concentration may differentiate low‐grade from non‐low‐grade PanNEN, with an AUC of 0.728.^16^

All available studies in the context of PanNEN are retrospective, and protocols vary substantially. Corrias et al. investigated intrinsic and extrinsic factors in iodine accumulation and their effect on final iodine concentration measurements.^27^ Their findings suggest deviations in injection rate and body mass index can profoundly influence iodine concentration. Additionally, contrast phase and measurement methods varied in recent literature concerning iodine concentration of pancreatic NETs. Despite preliminary evidence suggesting correlation between iodine concentration and tumor grade, the underlying data originate from heterogeneous, partly insufficiently documented measurement protocols, limiting meaningful cross‐study comparisons.

The observed positive correlation between Ki‐67 and RIC is intriguing but requires further investigation into its underlying mechanisms. Tumor angiogenesis may contribute to this relationship; however, additional trials are necessary to elucidate the exact biological pathways involved. A deeper understanding of these mechanisms could provide valuable insights for future therapeutic strategies.

PCCT systems can generate iodine maps superimposed with virtual non‐contrast imaging maps of contrast‐enhanced scans with minimal input required from the interpreting radiologist. As the necessary data are systematically acquired by design, the approach is applicable retrospectively as well. PCCTs offer a generational leap in imaging technology, with improvements in spectral imaging capabilities, further emphasizing the need to revisit iodine‐based biomarkers in a standardized manner. Our study focused on images in the portal venous phase, to minimize injection rate‐related variability. Moreover, our methodology is comprehensively defined and reported, facilitating future comparability.

Clinically, a non‐invasive tool to estimate tumor grading and particularly to distinguish high‐grade from low‐grade panNEN could support therapeutic stratification and guide surveillance in small non‐functional pancreatic NET as addressed in the ASPEN‐ trial.^4^ A change in RIC towards higher tumor grade could serve as an additional parameter defining the timing of surgery or prompt biopsy. To date, only the change in tumor size or onset of symptoms is considered a robust parameter for switching from watchful waiting to active therapy in non‐functional panNEN up to 2 cm.^3^, ^28^

Distinguishing NET G3 from NEC with low Ki67 (<55%) is a major challenge in clinical practice, as treatment strategies and tumor biology differ substantially, while histopathological diagnosis is not trivial.^4^ NEC's are generally more aggressive and NET G3 do not respond well to platinum‐based chemotherapy.^28^, ^29^ Immunohistological and molecular markers, such as DAXX and ATRX expression in NET G3 or p53 and RB‐1 alterations in NEC, can support accurate diagnosis.^30^, ^31^ Whether RIC has the potential to contribute to differentiation between these clinically challenging entities remains to be investigated in future studies. Another important aspect to be explored is the correlation between RIC and tumor vessel density, which may have therapeutic implications as well.

Within the limitations of this study, our findings are largely consistent with most previous literature. While ICC for RIC was highest (0.827), this should not be overinterpreted as an indicator of the parameters' superiority over IC and NIC, as 95% confidence intervals overlapped. RIC showed a statistically significant and strong positive correlation with Ki67 and tumor grade, whereas NIC showed only mild, non‐significant correlations, and IC showed slight non‐significant negative correlations. Furthermore, mean RIC in high‐grade PanNEN (G3/4) was significantly higher. Unfortunately, our cohort is too small to allow robust receiver‐operating characteristic and precise estimates of test performance.

This study has several limitations. First, it is designed as a retrospective single‐center pilot study. Second, the patient cohort is small. Further investigations, ideally in a multicentric setting with larger cohorts and standardized protocols, are needed to confirm our promising findings.

In conclusion, our preliminary results suggest that iodine concentration, particularly relative iodine concentration (RIC), may be a promising, non‐invasive biomarker for tumor grade and Ki67 prediction, with strong and statistically significant correlations respectively in PanNEN.

## AUTHOR CONTRIBUTIONS

**Marwin‐Jonathan Sähn:** Investigation; writing – original draft; visualization. **Marcel Bähr:** Methodology; formal analysis; resources; software.

## FUNDING INFORMATION

This study has not received third‐party funding.

## CONFLICT OF INTEREST STATEMENT

The authors declare no conflicts of interest.

## ETHICS STATEMENT

This retrospective study was approved by the Ethics Committee of the Faculty of Medicine, Ruhr‐University Bochum (2021‐827).

## PATIENT CONSENT STATEMENT

Due to the retrospective nature of this study, the need for informed consent was waived.

## Supporting information


**Data S1.** Supporting Information.

## Data Availability

The data that support the findings of this study are available on request from the corresponding author. The data are not publicly available due to privacy or ethical restrictions.
