# Supplementary material for: Relative and normalized iodine concentrations derived from photon counting computed tomography and their correlation with tumor grade and Ki67 in pancreatic neuroendocrine neoplasia: A pilot study
Source: J Neuroendocrinol. 2026 Feb 12;38(2):e70137. doi: 10.1111/jne.70137 (PMC12895385; doi:10.1111/jne.70137)
Supplement: Supplementary file 1 — Data S1. Supporting Information. [file JNE-38-e70137-s001.docx]

**Supplementary Information**Methods

Tube voltage was 120kVp, with tube current modulated based on a proprietary dose modulation with set image quality level (IQ level) 170. Total collimation was 144×0.4 mm, pitch factor was 0.8 and gantry rotation time was set to 0.5 s. Scan data sets were reconstructed with Br36 (polyenergetic) and Qr36 (spectral data set) convolution kernels, with 4 iterations (Q4 Quantum Iterative Reconstruction). Virtual monoenergetic images, as well as Iodine concenration maps were calculated axially using proprietary software (SnygoVia, Siemens Healthineers, Erlangen, Germany). Slice thickness was set to 0.6 m and increment to 0.4 mm, with matrix set to 512 x 512 pixels.
